# Construction and functional analysis of ceRNA regulatory network related to the development of secondary hair follicles in Inner Mongolia cashmere goats

**DOI:** 10.3389/fvets.2022.959952

**Published:** 2022-08-25

**Authors:** Fangzheng Shang, Rong Ma, Youjun Rong, Jianfeng Pan, Min Wang, Shuran Niu, Yunpeng Qi, Yanbo Li, Zhiying Wang, Qi Lv, Ruijun Wang, Rui Su, Zhihong Liu, Yanhong Zhao, Zhixin Wang, Jinquan Li, Yanjun Zhang

**Affiliations:** ^1^College of Animal Science, Inner Mongolia Agricultural University, Hohhot, China; ^2^Key Laboratory of Mutton Sheep Genetics and Breeding, Ministry of Agriculture, Hohhot, China; ^3^Key Laboratory of Animal Genetics, Breeding and Reproduction, Inner Mongolia Autonomous Region, Hohhot, China; ^4^Engineering Research Center for Goat Genetics and Breeding, Inner Mongolia Autonomous Region, Hohhot, China

**Keywords:** cashmere goats, secondary hair follicle, circular RNA, competing endogenous RNAs, functional analysis

## Abstract

Cashmere goat hair follicles are divided into primary hair follicles and secondary hair follicles. The primary hair follicles produce coarse hair, and the secondary hair follicles produce cashmere. The development of hair follicles is affected by a variety of signaling molecules and pathways. Studies have shown that non-coding RNAs are widely involved in the development of hair follicles of the goat, including small RNAs (miRNAs), long non-coding RNAs (lncRNA), and circular RNAs (circRNAs). In recent years, circRNAs, as a new type of circular closed non-coding RNAs, have attracted great attention due to their high stability. However, its regulatory effect on cashmere goat hair follicles mainly focuses on the periodic regulation of secondary hair follicles, and there is no report on the development of cashmere goat hair follicles during the fetal period. Therefore, this study was based on the circRNA, miRNA, and mRNA expression profiles obtained by whole-transcriptional sequencing of the skin tissue of the Inner Mongolia cashmere goats in the fetal period (days 45, 55, 65, and 75) and screening out the morphological changes of hair follicles at different periods. A total of 113 circRNAs related to the development of secondary hair follicles were present. According to the principle of the ceRNA regulatory network, a ceRNA regulatory network composed of 13 circRNAs, 21 miRNAs, and 110 mRNAs related to the development of secondary hair follicles was constructed. Then, qRT-PCR and Sanger sequencing identified circRNA2034, circRNA5712, circRNA888, and circRNA9127 were circRNAs. Next, the dual-luciferase reporter gene verified the targeting relationship of circRNA5712-miR-27b-3p-Dll4. In conclusion, this study constructed a ceRNA regulatory network for the development of cashmere goat secondary hair follicles, laying a foundation for the analysis of circRNAs regulating the morphogenesis and development of cashmere goat secondary hair follicles through the ceRNA mechanism.

## Introduction

Inner Mongolia cashmere goat is a unique local breed formed by natural selection and artificial selection for a long time. The cashmere produced by it is white and soft and is known as “fiber gem” and “soft gold.” Cashmere goats belong to heterogeneous fur animals, including primary hair follicles and secondary hair follicles. The primary hair follicles produce coarse hair, and the secondary hair follicles produce cashmere. Studies have shown that the initiation of morphogenesis of the primary hair follicles and the secondary hair follicles in cashmere goats is during different stages of fetal development, and the initiation of primary hair follicles is earlier than that of secondary hair follicles (1). During the 45–55 days of the fetal period, the skin forms a complete epidermal structure, and the hair follicles are not yet developed. During the 55–65 days of the fetal period, the primary hair follicles in various parts of the fetus begin to develop. On the 65th day, obvious primary hair follicle hair buds can be observed on the side of the body. From 65 to 75 days, secondary hair follicle primitives could be observed in various parts of the fetus, and secondary hair follicles began to occur. At 75 days, obvious secondary hair follicle hair buds could be observed on the side of the body (Supplementary Figure 1) (2, 3).

miRNAs are a class of non-coding RNAs of about 22 nt in length (4, 5), which usually target the 3'UTR of genes to act as post-transcriptional negative regulatory elements of gene expression, thereby affecting several major biological pathways (6, 7). The research of some non-coding RNAs in hair follicles has also made great progress. Studies have found that miRNA-203 can regulate the hair follicle development of cashmere goats by targeting DDOT and NAE1 (8).

chi-miR-370-3p can inhibit the development of cashmere goat hair follicle epithelial cells and dermis by targeting TGF-βR2 and FGFR2. The proliferation of fibroblasts and increase their cell migration ability (9), chi-miR-199a-5p and chi-miR-200a regulate the occurrence and development of the hair follicle by targeting TGF-β2 (10).

CircRNAs are a newly discovered class of endogenous non-coding RNAs, which are single-stranded closed circular structures produced by end-to-end back-splicing of the 3' and 5' ends of mRNA precursors (11). Unlike linear RNAs, circRNAs are resistant to the influence of exonuclease due to a lack of 5' cap structure and 3' poly(A) tail and are more stably expressed than most linear RNAs (12, 13). They usually have extremely abundant miRNA binding sites and can act as excellent molecular sponges (14). CeRNA was first proposed by the Pandolfi team of Harvard Medical School in 2011. These ceRNA molecules (including circRNA, lncRNA, mRNA, pseudogene, etc.) can competitively bind to the same miRNA through miRNA response elements (MREs), thereby regulating the expression levels of each other (15). In recent years, many scholars have verified that circRNAs and lncRNAs regulate hair follicle growth through the ceRNA mechanism. Studies have found that lncRNA-XIST acts as a miR-424 sponge to promote the expression of Shh, thereby activating hedgehog signaling to promote dermal papilla-mediated hair follicle regeneration (16), while lncRNA-PCAT1 activates the Wnt/β-catenin signaling pathway by targeting miR-329 to maintain the biological characteristics of dermal papilla cells and promote hair follicle regeneration (17). In addition, circFTO and circCSPP1 can competitively bind miR-148a and miR-10a to enhance BMP7 expression and promote the proliferation of lake wool papilla cells (18), and circRNA-1967 can increase the expression of LEF1 by adsorbing miR-93-3p that regulated the differentiation of cashmere goat secondary hair follicle stem cells to the hair follicle lineage (19).

However, the molecular mechanism by which circRNA regulates the development of secondary hair follicles in the fetal stage of the cashmere goat through ceRNA remains unclear. Therefore, in this study, we screened circRNAs related to the development of secondary hair follicles in cashmere goats. Through bioinformatics prediction, combined with the screening results of miRNAs related to the development of secondary hair follicles in the early stage and the pathway analysis of miRNA target genes, the development of secondary hair follicles in cashmere goats was constructed. The related ceRNA regulatory network laid the foundation for the subsequent in-depth analysis of the mechanism of circRNA regulating the development of cashmere goat's secondary hair follicles through the ceRNA mechanism.

## Materials and methods

### Sample

The test samples were collected from Inner Mongolia Jinlai Animal Husbandry Technology Co., Ltd. The production ewes were used to form an experimental group, the mating time was recorded, and the flank skin samples of cashmere goat fetuses at days 45, 55, 65, and 75 of pregnancy were collected through cesarean section (all fetal skin samples were in accordance with the international guidelines for animal research) and approved by the Scientific Research and Academic Ethics Committee of Inner Mongolia Agricultural University and the Biomedical Research Ethics of Inner Mongolia Agricultural University (Approval No. [2020] 056)). Three fetal skin samples per period were used as biological replicates, and the collected skin was washed with DEPC water and numbered, immediately. They were first placed in liquid nitrogen and then in a −80°C refrigerator for future use.

### Analysis of differential circRNAs in each comparison group

According to the expression of circRNAs, the differentially expressed circRNAs in each comparison group were analyzed, and the screening criteria for differences were that the expression level changed more than 2 times and the *p*-value was <0.05.

### Screening of circRNAs related to the development of secondary hair follicles

According to the hair follicle morphogenesis law of a previous research, days 65–75 are the dividing point of the primary hair follicle and secondary hair follicle development. The differentially expressed circRNAs of stage A (day 65 vs. 45, day 65 vs. 55, and day 55 vs. 45) are used as the circRNA related to primary hair follicle development, and the differentially expressed circRNAs of Stage B are (day 75 vs. 45, day 75 vs. 55, and day 75 vs. 65) are used as the circRNA that jointly affects primary hair follicle and secondary hair follicle development. Stage A and Stage B are intersected to obtain the circRNA that is related to secondary hair follicle development.

### Prediction of circRNA targeted miRNAs and key miRNA targeted genes

We used TargetScan and miRanda to predict miRNAs targeted by circRNAs. TargetScan predicted miRNA targets based on the seed region. miRanda was mainly based on the binding free energy of circRNA and miRNA. The smaller the free energy, the better the binding ability of the two, which is powerful (The value of TargetScan and miRanda is analyzed, and the TargetScan threshold is 50. The larger the value, the greater the possibility of interaction between the two. The miRanda threshold is −10. The smaller the value, the more likely the interaction between the two is large) to obtain the circRNA-miRNA targeting relationship. Similarly, the target genes of key miRNAs are predicted to obtain the miRNA-mRNA targeting relationship.

### Construction of ceRNA regulatory network related to secondary hair follicle genesis in cashmere goats

Based on the above research results, the key miRNAs were obtained by intersecting the miRNAs targeted by circRNAs and the miRNAs related to the development of secondary hair follicles screened in the previous stage, and combined with the enrichment analysis results of key miRNA target genes. The ceRNA regulatory network related to the development of secondary hair follicles was constructed.

### qRT-PCR

The expression of four key circRNAs in the skin hair follicles of the cashmere goat embryo at different stages was detected by qRT-PCR. Total RNA was treated with R Nase R (RNA exonuclease) in a water bath at 37°C for 25 min and a water bath at 70°C for 10 min to remove the linear RNA. Then, the RNA was reverse transcribed using TaKaRa's PrimeScript™ RT reagent Kit with the gDNA Eraser reverse transcription kit. The Roche LightCycler^®^ 96 real-time quantitative PCR instrument was used, the reaction volume was 20 μL, and β-actin was used as the internal reference (20). Then, the relative expression was calculated using the 2^−ΔΔ^CT method.

### Identification of key circRNAs in ceRNA regulatory networks

The structural composition of key circRNAs was determined by bioinformatics, and then the identification primers were designed and synthesized. PCR amplification and agarose gel electrophoresis Sanger sequencing were performed using cashmere goat fetal skin cDNA as a template to determine whether the circularization site was amplified.

### Functional analysis

The chi-miR-27b-3p mimic was synthesized by Shanghai Han Biotechnology Co., Ltd., China. The psiCHECK2-circRNA5712-WT and psiCHECK2-DLL4-WT constructs were generated by inserting circRNA5712 and DLL4 fragments containing miRNA-binding sequences into the psiCHECK-2 vector (Promega) at the 3′ end of the Renilla luciferase gene, and psiCHECK2-circRNA5712-MUT and psiCHECK2-DLL4-MUT structures were generated by mutating the miRNA-binding sequence to a complementary sequence. For DLL4 luciferase assay, HEK 293T cells were transfected with miRNA mimic and psiCHECK2-circRNA5712-WT (psiCHECK2-DLL4-WT) or mutant psiCHECK2-circRNA5712-Mut (psiCHECK2-DLL4-Mut) reporter plasmid. Luciferase activity was measured 48h after transfection using a dual-luciferase reporter detection system (Promega). The relative luciferase activity was calculated by comparing the ratio of firefly/nephro luciferase activity.

Operation steps of cell transfection:

1. A total of 293T cells and target plasmids were prepared for transfection into 96 well plates in advance followed by a waiting period until the cell density reaches 50–70%.
2. Next, 10 μl DMEM was mixed with 0.16 μg h-HDAC1-3UTR target plasmid, 5pmol hsa-miR-449a/negative Control (N.C) is fully mixed and placed at room temperature (solution A), and then 10 μl DMEM is fully mixed with 0.3 μl transfection reagent (the transfection reagent is hanheng biological product, and the concentration is 0.8 mg/ml) (solution B) and placed at room temperature for 5 min.
3. Solution A and solution B were fully mixed and placed at room temperature for 20 min.
4. Fresh culture medium was exchanged for cells before transfection, and then the transfection mixture was added and mixed at 37°C in a 5% CO_2_ culture.
5. Fresh culture medium was exchanged 6 h after transfection, and cells for detection were collected 48 h after transfection.

## Results

### Differentially expressed circRNAs in each comparison group

**Figure 1:**
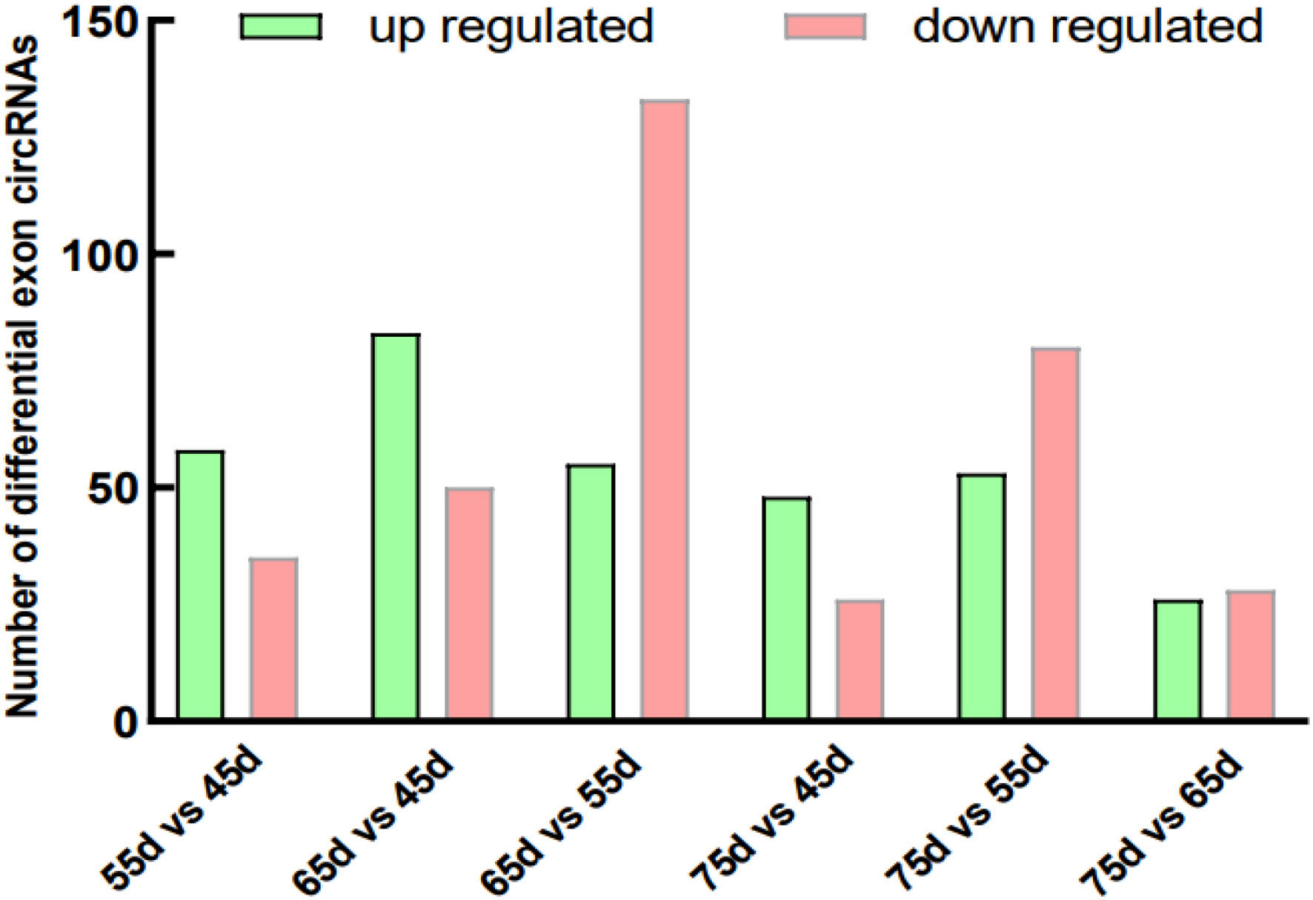
Differentially exon circRNAs in different groups. The green column represents upregulated circRNA and the pink column represents downregulated.

The skin tissue of Inner Mongolia cashmere goat at different embryonic stages (days 45, 55, 65, and 75) formed six comparison groups (day 75 vs. 45, day 75 vs. 55, day 75 vs. 65, day 65 vs. 55, day 65 vs. 45, and day 55 vs. 45). CircRNAs are mainly divided into four categories: exonic circRNAs, exon-intron circRNAs, intronic circRNAs, and intergenic circRNAs. This study focused on the exploration of exonic circRNAs. The results counted the number of differentially expressed exonic circRNAs in each comparison group (Figure 1) and the number of other types of differentially expressed circRNAs (Supplementary Figure 2). The number of differentially expressed exon circRNAs are: day 55 vs. 45 upregulated 58 and downregulated 35; day 65 vs. 45 upregulated 83 and downregulated 50; day 65 vs. 55 upregulated 55 and downregulated 133; day 75 vs. 45 upregulated 48 and downregulated 26; day 75 vs. 55 upregulated 53 and downregulated 80; and day 75 vs. 65 increased by 26 and decreased by 28. This lays the foundation for the subsequent screening of circRNAs related to the development of secondary hair follicles in cashmere goats.

### circRNAs related to the development of secondary hair follicles

**Figure 2:**
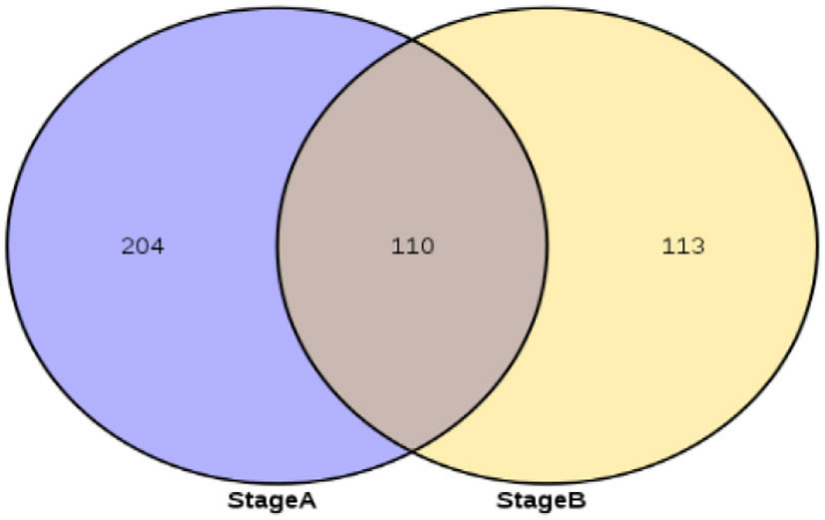
circRNAs associated with secondary hair follicle morphogenesis and development.

Stage A of secondary hair follicle morphogenesis is (day 65 vs. 45, day 65 vs. 55, and day 55 vs. 45) differentially expressed exonic circRNAs and StageB is (day 75 vs. 45, day 75 vs. 55, and day 75 vs. 65) differentially expressed exonic circRNAs. The results showed that the number of differential circRNAs in Stage A was 314, and the number of differential circRNAs in Stage B was 223. The 110 differential circRNAs shared by Stage B and Stage A were screened out, and the remaining 113 circRNAs were used as circRNAs related to the development of secondary hair follicles (Figure 2).

### Prediction of circRNA targeted miRNAs related to the development of secondary hair follicles

**Figure 3:**
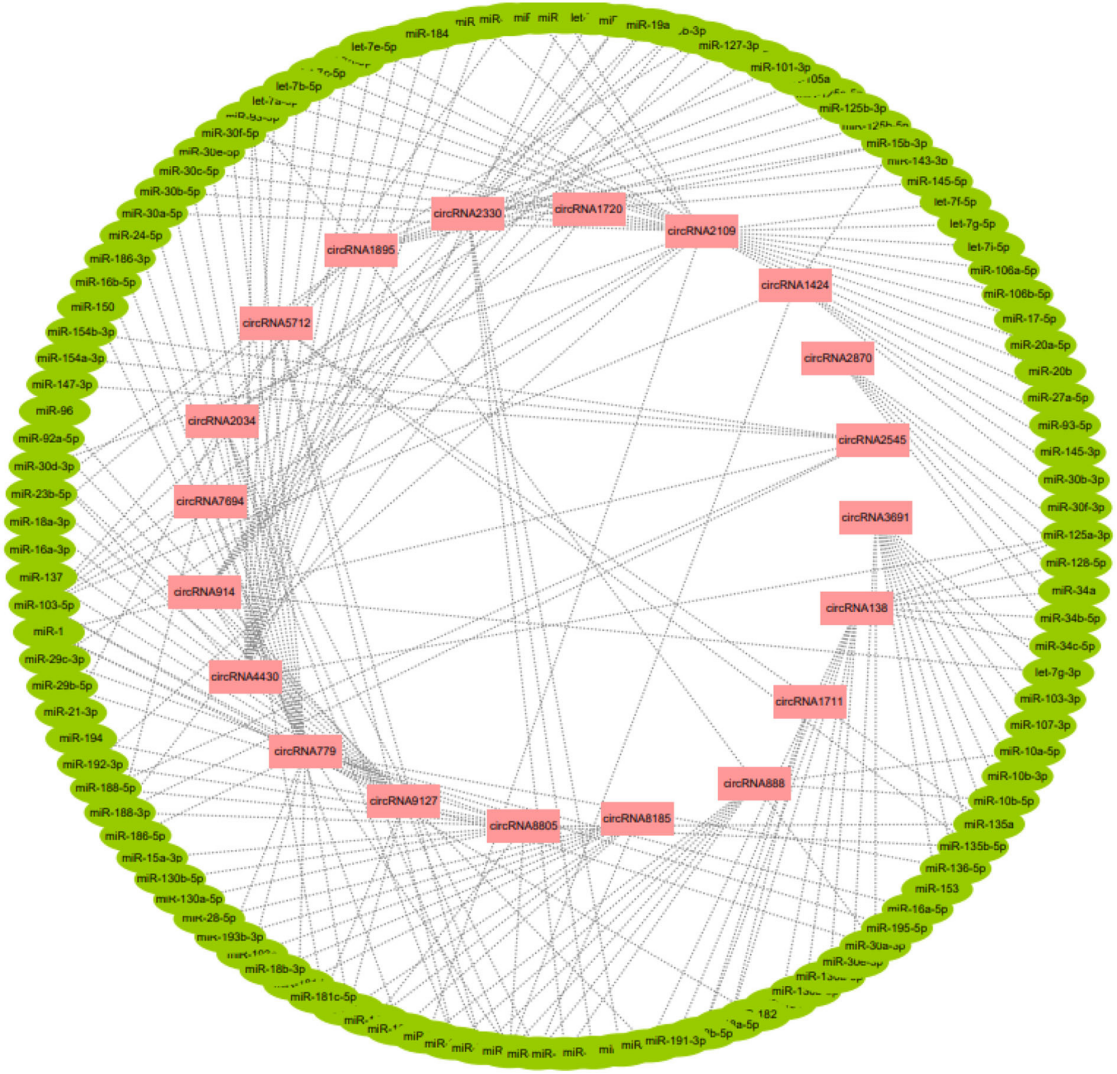
The regulatory network of circRNA-miRNA related to secondary hair follicle development.

A total of 20 of the 113 circRNAs related to the development of secondary hair follicles screened above were selected, and their length, host gene, and exon number were obtained by bioinformatics analysis. It was found that all circRNAs were less than 2,000 bp in length and consisted of one or more exons (Table 1). At the same time, its target miRNAs were predicted, 117 target miRNAs were obtained, and 194 circRNA-miRNA regulatory networks related to the morphogenesis and development of secondary hair follicles were constructed (Figure 3).

**Table 1:** Information on circRNA related to secondary hair follicle development.

**Accession**	**CircRNA size**	**Hosting gene name**	**Exon count**	**CircRNA type**	**IsoformName**
circRNA2545	555	AKT3	4	ecircRNA	XM_018060261.1
circRNA2870	430	PALLD	2	ecircRNA	XM_018051789.1
circRNA3691	1107	PREX2	9	ecircRNA	XM_018058434.1
circRNA138	339	TRPS1	2	ecircRNA	XM_018058367.1
circRNA779	1615	PIK3CA	8	ecircRNA	XM_005675289.3
circRNA4430	1615	ITCH	14	ecircRNA	XM_018057738.1
circRNA914	349	RAP1B	4	ecircRNA	XM_018047553.1
circRNA7694	321	LRIG1	1	ecircRNA	XM_018066999.1
circRNA1711	303	RASA1	2	ecircRNA	XM_013965776.2
circRNA888	493	PLXNC1	3	ecircRNA	XM_013963727.2
circRNA8185	691	LTBP1	6	ecircRNA	XM_018055103.1
circRNA8805	1161	ACVR2A	8	ecircRNA	XM_005676173.3
circRNA1720	771	ZFYVE16	6	ecircRNA	XM_018050066.1
circRNA2034	836	ATE1	6	ecircRNA	XM_018041398.1
circRNA1895	1391	PARP8	11	ecircRNA	XM_018065625.1
circRNA2109	929	PCNX1	8	ecircRNA	XM_018054011.1
circRNA1424	942	ZNF638	6	ecircRNA	XM_018055075.1
circRNA5712	338	102172906	3	ecircRNA	XM_018055309.1
circRNA9127	1238	102183487	3	ecircRNA	XM_018042231.1
circRNA2330	590	DCUN1D4	7	ecircRNA	XM_018049448.1

### Screening of key miRNAs and their target gene prediction

**Figure 4:**
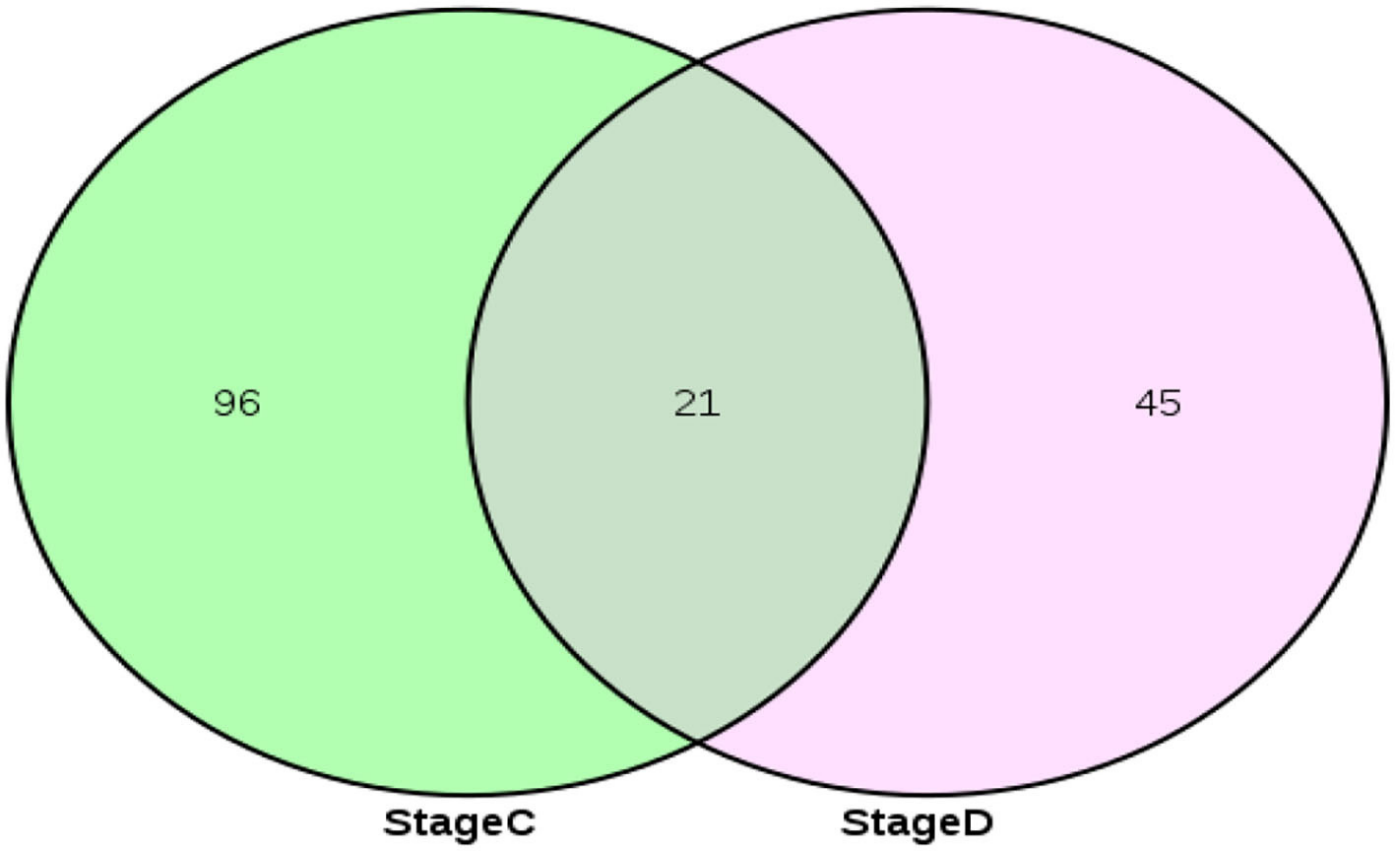
Screening of key miRNAs in ceRNA regulatory network related to secondary hair follicle development.

**Figure 5:**
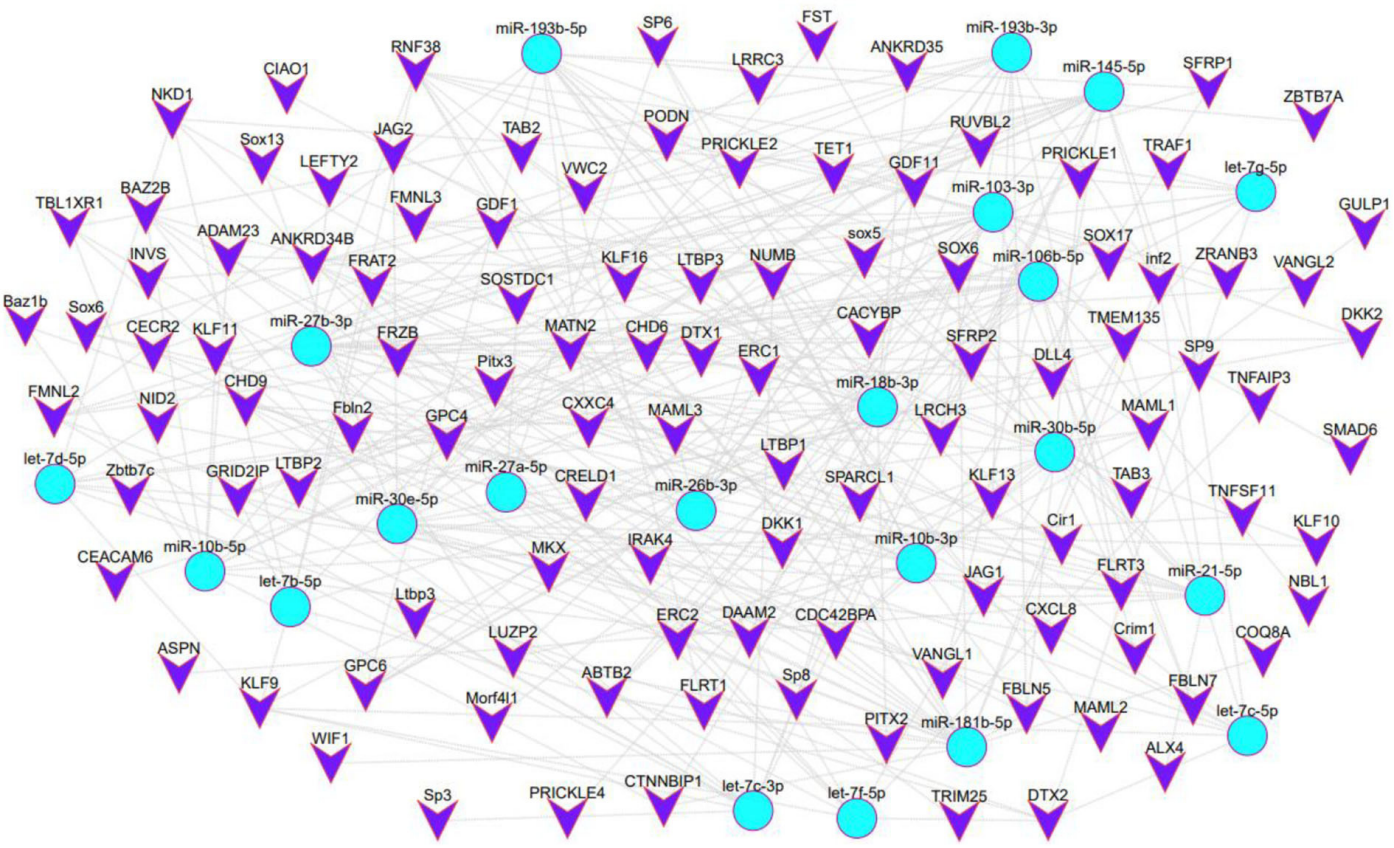
The regulatory network of miRNA-mRNA related to secondary hair follicle development.

The above predicted 117 targeted miRNAs were intersected with 66 miRNAs related to secondary development previously screened (21) to obtain 21 key miRNAs in the ceRNA regulatory network related to secondary follicle morphogenesis and development (Figure 4). Bioinformatics predicted its target genes, screened 110 target genes enriched in Wnt, TGF-β, Notch, and NF-Kappa B, and constructed 280 miRNA-mRNA regulatory networks related to the development of secondary follicles (Figure 5).

### Construction of a ceRNA regulatory network related to the development of secondary hair follicles

**Figure 6:**
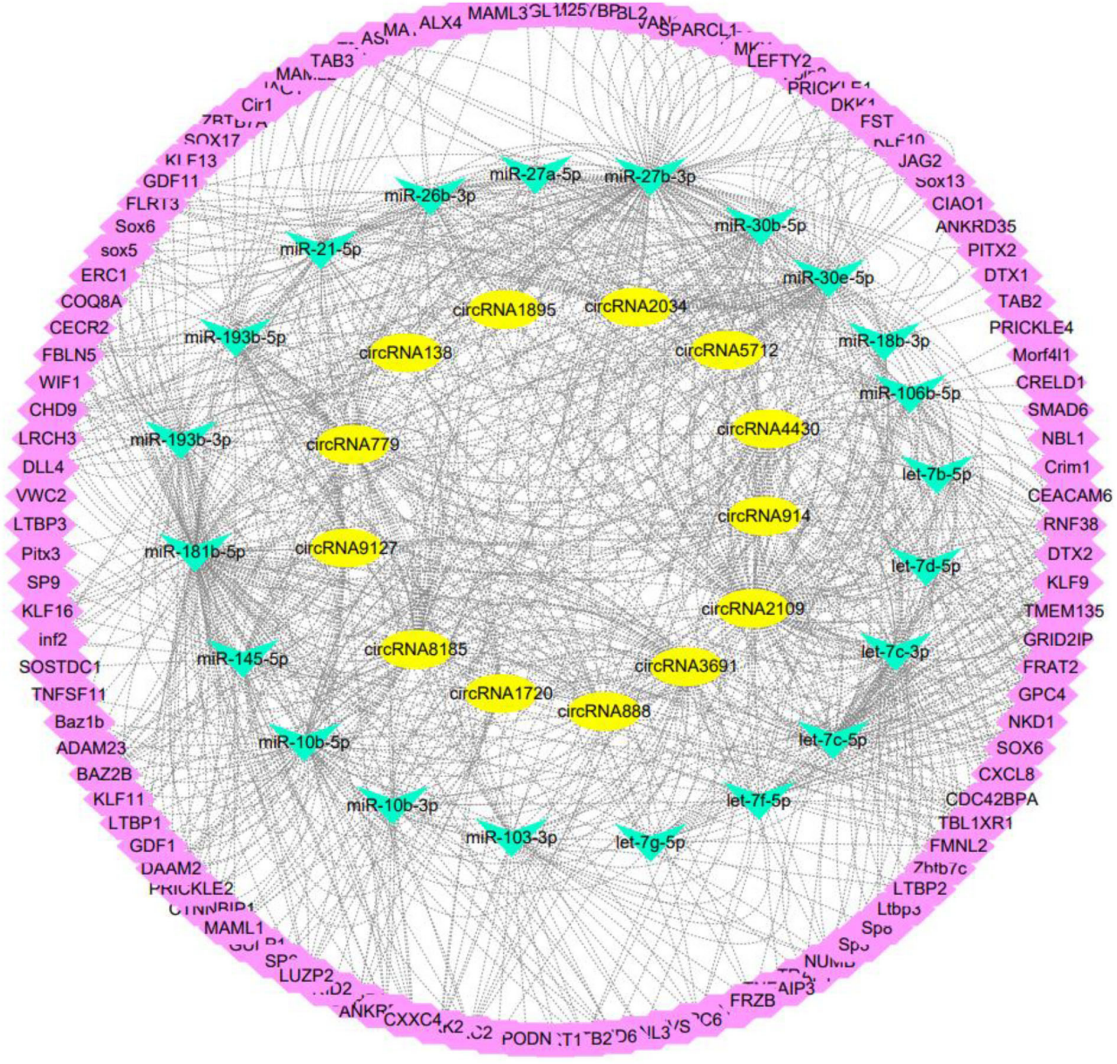
ceRNA regulatory network related to secondary hair follicle development.

Based on the circRNAs, miRNAs, and mRNAs screened above, this study constructed a ceRNA regulatory network related to the development of secondary hair follicles consisting of 13 circRNAs, 21 miRNAs, and 110 mRNAs, including circRNA5712-miR-27b-3p-DLL4, circRNA2109-miR-30e-5p-DKK1, circRNA9127-miR-30e-5p-FST, and circRNA3691-miR-103-3p-ERC2 as well as other 500 secondary hair follicles related to the development ceRNA regulatory network (Figure 6).

### Identification of circRNAs

**Figure 7:**
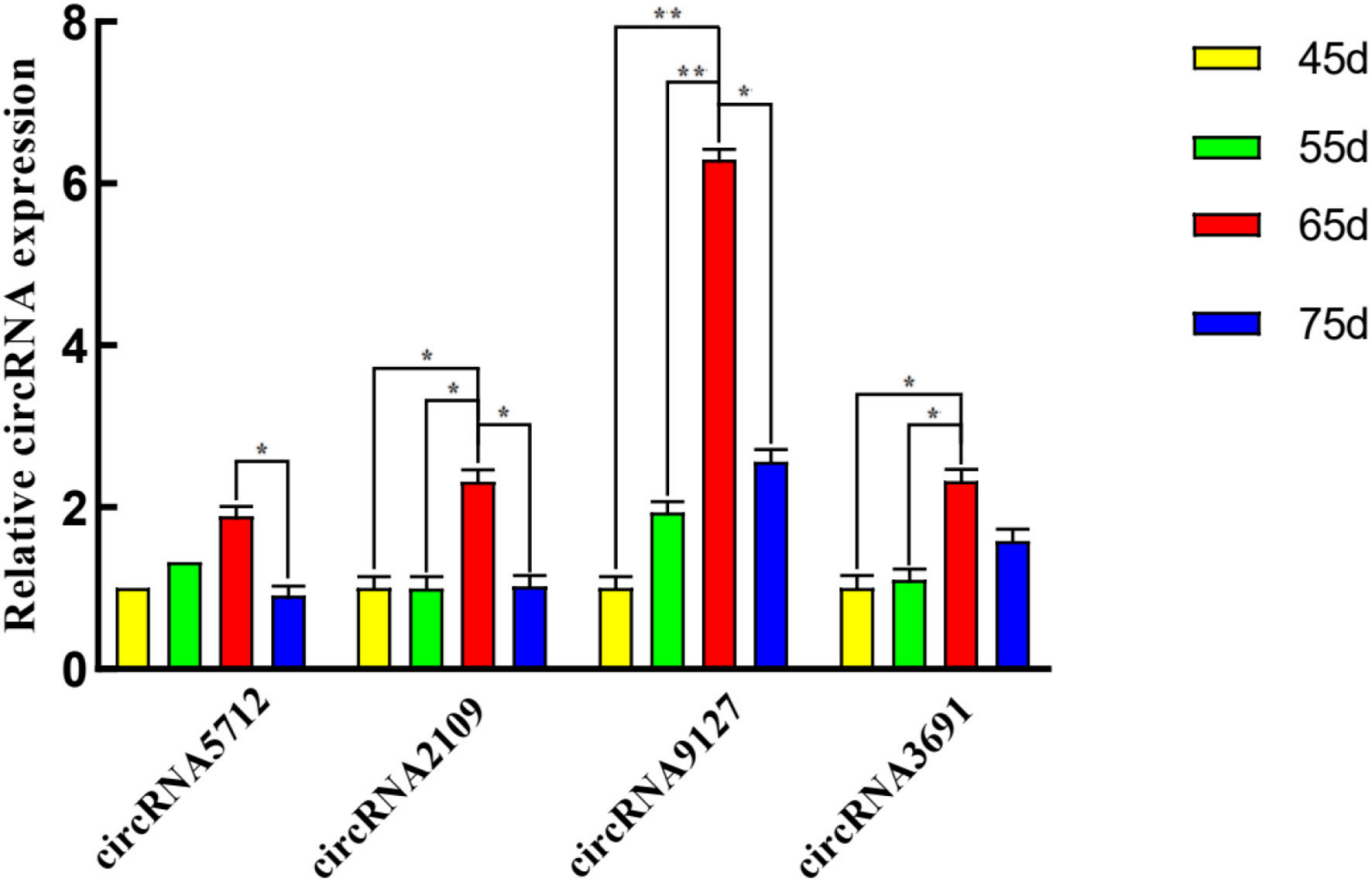
qRT-PCR was used to detect the expression of circRNAs in the skin of cashmere goats at different stages. The * symbol indicates the value of *P* < 0.05 with significant difference. The ** represents *P* < 0.01 with extremely significant difference.

**Figure 8:**
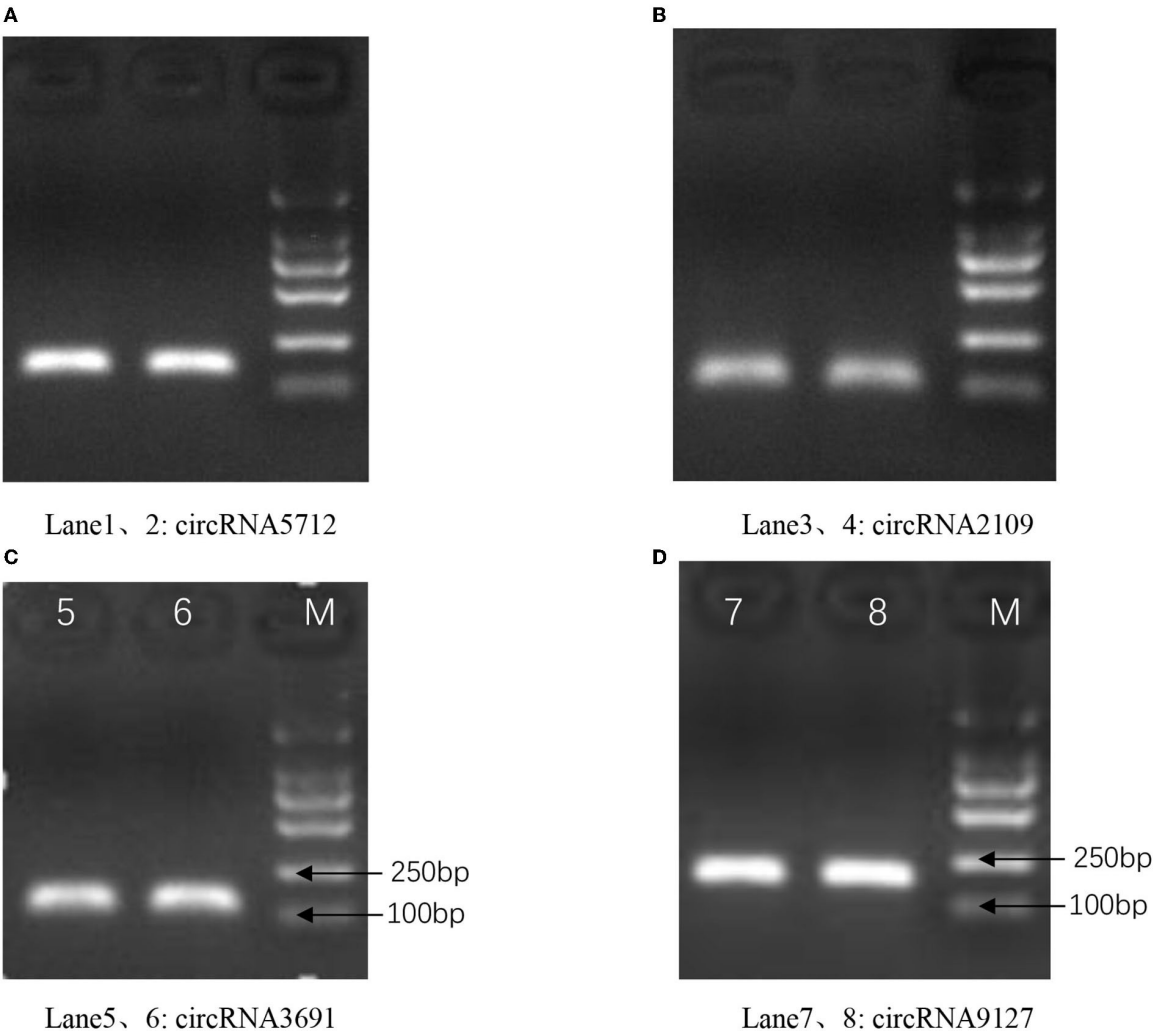
The existence of circRNA was confirmed by PCR and agarose gel electrophoresis assay. Panels **(A–D)** represent the amplified bands of circRNA5712, circRNA2109, circRNA9127, and circRNA3691 on agarose gel electrophoresis, respectively.

**Figure 9:**
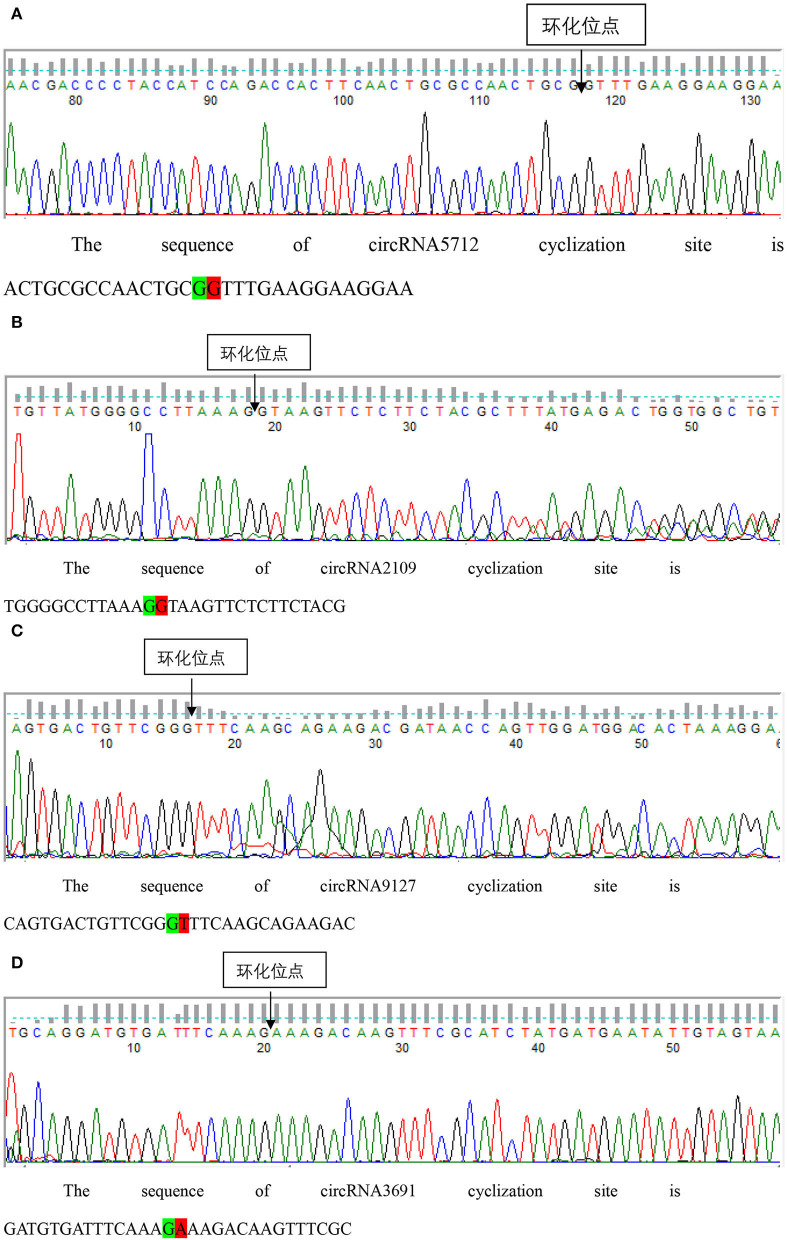
Sanger sequencing was used to detect the structure of each circRNA. Panels **(A–D)** represent the results of Sanger sequencing circRNA5127, circrna2109, circRNA9127, and circRNA3691, respectively.

The results of qRT-PCR experiments showed that four key circRNAs in the ceRNA regulatory network, circRNA5712, circRNA2109, circRNA9127, and circRNA3691, were expressed in the fetal skin of cashmere goats at different stages, and they were different (Figure 7). Bioinformatics analysis showed that circRNA5712 was 228 bp and consisted of the entire sequence of exons 3 to 5 of goat 102172906 gene, and circRNA2109 was 929 bp and consisted of the entire sequence of goat PCNX1 gene exons 9 to 16. The length of the sequence of circRNA9127 was 1,238 bp, which consists of the entire sequence of exons 2 to 4 of goat gene 102183487, and the length of circRNA3691 is 1,107 bp, which consists of the entire sequence of exons 4 to 12 of goat PREX2 gene (Table 2). By RNA degradation, PCR amplification, and agarose gel electrophoresis, it was found that the amplified bands of circRNA5712, circRNA2109, circRNA9127, and circRNA3691 were consistent with the target bands (Supplementary Figure 3 and Figure 8); Sanger sequencing showed that they all had cyclization sites (Figure 9). The above verification indicated the real existence of circRNA5712, circRNA2109, circRNA9127, and circRNA3691.

**Table 2:** The structural composition of key circRNA in the development of secondary hair follicles in cashmere goat.

**circRNA**	**circRNA size**	**Exon**	**Start**	**End**	**Length/bp**	**Host gene**
circRNA5712	338	Exon3	44323276	44323210	67	
		Exon4	44322571	44322451	121	102172906
		Exon5	44322101	44321952	150	
		Exon9	20483509	20483419	91	
		Exon10	20478862	20478805	58	
		Exon11	20477346	20477129	218	
		Exon12	20467888	20467735	154	
circRNA2109	929	Exon13	20464738	20464706	33	PCNX1
		Exon14	20463193	20463022	172	
		Exon15	20462600	20462490	111	
		Exon16	20459658	20459567	92	
		Exon2	1767838	1767904	67	
circRNA9127	1,238	Exon3	1768778	1769764	987	102183487
		Exon4	1770624	1770807	184	
		Exon4	50049085	50048981	105	
		Exon5	50043652	50043551	102	
		Exon6	50040370	50040209	162	
		Exon7	50029626	50029493	134	
circRNA3691	1,107	Exon8	50026337	50026234	104	PREX2
		Exon9	50024779	50024630	150	
		Exon10	50020604	50020460	145	
		Exon11	50015480	50015380	101	
		Exon12	50007891	50007788	104	

### Functional analysis of circRNA5712-miR-27b-3p-DLL4

**Figure 10:**
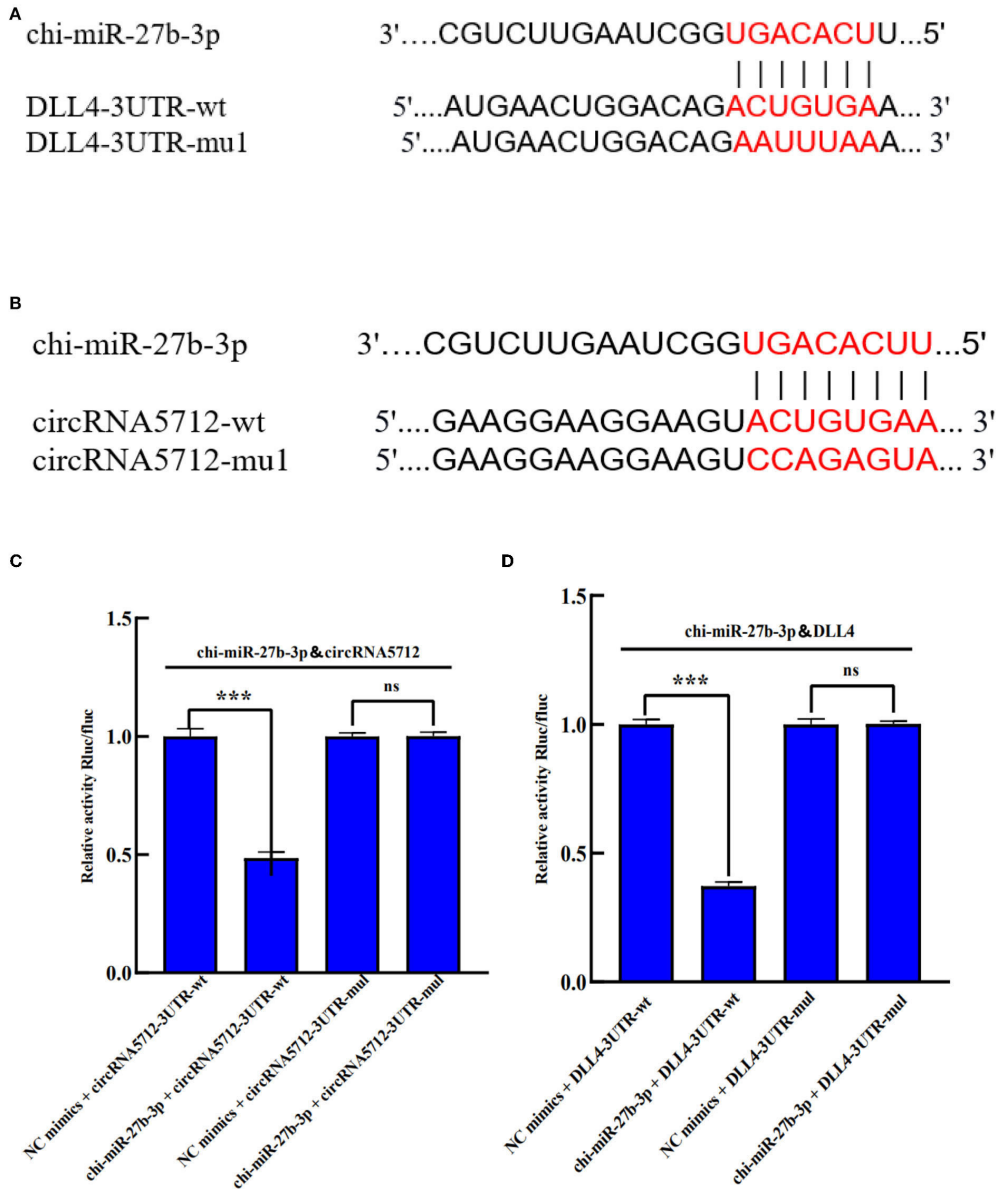
Verification of the targeting relationship between circRNA5712, DLL4 and chi-miR-27b-3p in Inner Mongolia cashmere goat. **(A)** chi-miR-27b-3p and circRNA5712 3'-UTR binding sites and mutation sites. **(B)** chi-miR-27b-3p and DLL4 3'-UTR binding sites and mutation sites. **(C)** Verification of the interaction between chi-miR-27b-3p and circRNA5712 3'-UTR detected by a dual-luciferase reporter gene assay (***P < 0.001). **(D)** Verification of the interaction between chi-miR-27b-3p and DLL4 3'-UTR detected by a dual-luciferase reporter gene assay (***P < 0.001). Results in **(C,D)** are expressed as mean 6 standard error of the mean (SEM).

The circRNA5712-miR-27b-3p-DLL4 in the ceRNA regulatory network constructed above was selected for functional verification. We used TargetScan and miRanda to predict the existence of binding sites for chi-miR-27b-3p with circRNA5712 and DLL4. Therefore, mutant vectors were constructed to verify specific binding sites (Figures 10A,B). The results showed that compared with the NC group, chi-miR-27b-3p significantly decreased the expression of luciferase in circRNA5712 cells (*p* < 0.001). This suggests that there is a binding site between chi-miR-27b-3p and circRNA5712. After mu1 mutation, chi-miR-27b-3p failed to downregulate the expression of luciferase in circRNA5712-mut1 (*p* > 0.05), indicating that the mutation was successful. It was verified that chi-miR-27b-3p could bind to circRNA5712. The results showed that compared with the NC group, chi-miR-27b-3p significantly decreased the expression of DLL4 cell luciferase (*p* < 0.001). This suggests that there is a binding site between chi-miR-27b-3p and DLL4. After mu1 mutation, chi-miR-27b-3p failed to downregulate the expression of luciferase in DLL4-mut1 (*p* > 0.05), indicating successful mutation. It was verified that chi-miR-27b-3p could bind to DLL4 (Figures 10C,D). It was further proved that circRNA5712-miR-27b-3p-DLL4 has a targeting relationship. It is speculated that circRNA5712 may affect the expression of DLL4 through the ceRNA mechanism to competitively bind to miR-27b-3p, thereby regulating the development of cashmere goat secondary hair follicles.

### The model of ceRNA regulation related to secondary hair follicle development

**Figure 11:**
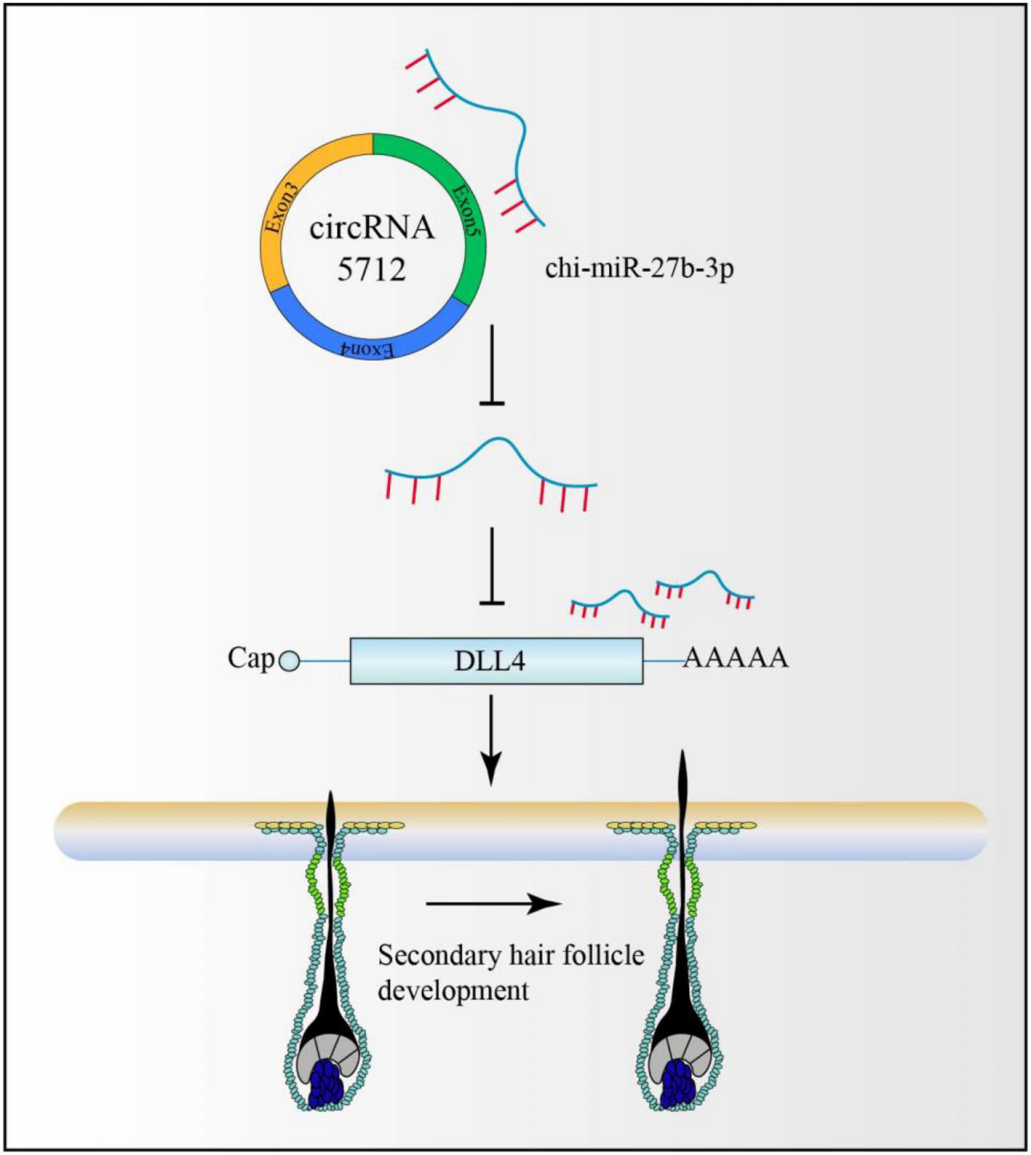
circRNA-miRNA-mRNA-secondary hair follicle model.

CircRNAs are non-coding RNAs that have been studied more in recent years. Some circular RNAs have MREs (a sequence recognized by miRNAs). Therefore, circRNAs mainly regulate mRNA expression by competing for binding to miRNAs and then participate in the regulation of various biological activities. On this basis, a cirRNA-miRNA-mRNA secondary hair follicle model was constructed (Figure 11), which provided a theoretical basis for the subsequent analysis of the molecular regulation mechanism of cirRNA in secondary hair follicles.

## Discussion

As circRNAs have become an important research object in the field of biology, their many regulatory roles have been gradually explored. In this study, the circRNA, miRNA, and mRNA expression profiles were obtained from the fetal skin tissue of cashmere goat at different stages (days 45, 55, 65, and 75) by whole-transcriptional sequencing, and the miRNA and mRNA related to the development of secondary hair follicles were screened. After identifying the differentially expressed circRNAs in each comparison group, combined with the morphological changes of cashmere goat secondary hair follicles in different periods, circRNAs related to the development of secondary hair follicles were screened. In recent years, researchers discovered circRNA1967, circRNA1926, and circRNA-0100 in cashmere goat secondary hair follicles and verified their regulatory roles in secondary hair follicles (19, 22, 23). In this study, 113 circRNAs related to the development of secondary hair follicles, including circRNA5712, circRNA2109, circRNA9127, and circRNA3691, were screened. It laid the foundation for the subsequent construction of the ceRNA network related to the development of secondary hair follicles.

Then, we used TargetScan and miRanda to predict 20 circRNAs related to the development of miRNAs including miR-30b-5p, miR-103-3p, and miR-106b-5p b of secondary hair follicles targeted miRNAs and obtained 21 keys intersecting the miRNAs that were screened in the previous stage. Previous studies have found that chi-miR-30b-5p plays an important role in regulating the cashmere goats (24), miR-103-3p regulates the periodic growth of hair follicles of Liaoning hair follicles (25), and miR-106b-5p may affect the development of hair follicles in cashmere goats during the fetal period (10). It indicated that the screening method of key miRNAs in the ceRNA regulatory network related to the development of secondary hair follicles is reasonable and reliable. Then, the target genes of 21 key miRNAs were predicted. According to the principle of combining miRNA and mRNA, there are usually many target genes. Therefore, we selected 110 target genes in the signaling pathway related to hair follicle development as the target genes of key miRNAs, such as DKK2, SFRP1, and SFRP2 in the WNT signaling pathway, FST and FBLN7 in the TGF-β signaling pathway, RNF38 and DLL4 in the Notch signaling pathway, and TNFRSF11A and ERC2 in the NF-KB signaling pathway. Numerous studies have shown that the development of hair follicles is mediated by a series of signaling pathways and related molecules. Wnt signaling is expressed in the development of hair follicle placodes, embryonic epidermis, and hair (26). Generally, the Wnt/β-catenin signaling pathway plays a key role in hair follicle morphogenesis and cyclization during embryonic and adulthood (27, 28). The addition of Wnt signaling in dermal papilla cells also stimulates hair growth (29). The TGF-β signaling pathway is involved in the development of the epidermis, the formation of the hair follicle placode, and the periodic growth of the hair follicle (30). Studies have found that TGF-β1 has a regulatory effect on the degenerative phase of hair follicles in mice. Knockout of TGF-β1 in mice significantly delayed the transition from the growth phase to the degenerative phase (31). TGF-β2 can inhibit hair shaft elongation and induce the hair follicle degenerative phase. During morphological changes (32), the Notch signaling pathway is involved in the early stages of hair follicle development and the differentiation of hair shafts in the later stage. Notch1 is mainly expressed in the hair follicle placode in the mouse embryonic stage. Ectopic expression of DLL1 can promote the expression of Notch1 and Notch2 and accelerate the expression of placode formation (33). NF/kB signaling can enhance the motility of epithelial cells and can increase the epithelial cells required to form the hair follicle placode (34). In addition, studies have found that molecules such as EDAR (35–37), Dkk4 (38), and Lhx2 (39) are involved in the differentiation of epithelial cells and the aggregation of dermal fibroblasts. DKK2 affects the development of hair follicles through Wnt/β-catenin and regulates hair or not hair traits (40). SFRP1 and SFRP2 genes are differentially expressed in sheep fetal skin tissue at different stages (41). FST is located in the hair follicle matrix and affects the quality of cashmere (42).

More and more studies have shown that non-coding RNAs play an important role in the development of hair follicles, among which the mechanism of competition for endogenous RNA (ceRNA) is more deeply studied. The ceRNA mechanism, that is the non-coding RNA competes with miRNA target genes to bind miRNA through its own miRNA response element (MRE), thereby indirectly regulating the process of miRNA target genes at the post-transcriptional level. Based on the above precise screening and prediction results, a ceRNA regulatory network for secondary hair follicle development was constructed consisting of 13 circRNAs, 21 miRNAs, and 110 mRNAs. Hui constructed a ceRNA regulatory network related to the fineness of cashmere (43), Liu constructed a ceRNA regulatory network of dairy goat endometrial epithelial cells (44), and Yue constructed a ceRNA regulatory network related to skeletal muscle development in Qinchuan cattle (45). All of them provide an important basis for exploring the functional role of circRNA/lncRNA through the ceRNA mechanism. Furthermore, some cricRNAs in the ceRNA regulatory network were identified, and the true existence of circRNA5712, circRNA2109, circRNA9127, and circRNA3691 was identified by qRT-PCR, PCR amplification, agarose gel electrophoresis, and Sanger sequencing. Hao et al. identified the authenticity of circRNAs such as circ_001091, circ_001082, and circ_002556 in cashmere goat mammary glands (46). Li et al. identified the authenticity of circRNAs such as circRNA_026259 and circRNA_024949 in Tibetan sheep testis (47), for further exploration of these circRNAs Their functional mechanism provides an important guarantee.

Finally, we functionally analyzed circRNA5712-chi-miR-27b-3p-Dll4 in the ceRNA regulatory network related to secondary hair follicle development. Through the verification of the double luciferase reporter gene, we found that chi-miR-27b-3p has a targeted relationship with circRNA5712 and Dll4, indicating that circRNA5712 affects the expression of Dll4 through chi-miR-27b-3p, thereby regulating the morphogenesis of secondary hair follicles.

In a word, at the initial stage of hair follicle development, primary hair follicles and secondary hair follicles are difficult to separate mechanically. Therefore, in order to explore the key signal molecules that affect secondary hair follicle development, this study tries to combine the histomorphology of different embryonic stages to screen the key circRNA of secondary hair follicle development in cashmere goats from the level of histomics data and construct the ceRNA regulatory network. However, the location of specific circRNA and its regulatory pathway still need to be verified by a large number of subsequent molecular experiments.

## Conclusion

Based on the circRNA expression profiles of cashmere goat skin at days 45, 55, 65, and 75, this study identified differentially expressed exonic circRNAs and other types of circRNAs and screened for secondary hair follicle development-related circRNAs. circRNA, and then constructed circRNA5712-miR-27b-3p-DLL4, circRNA2109-miR-30e-5p-DKK1, circRNA9127-miR-30e-5p-FST, and circRNA3691-miR-103-3p-ERC2 through prediction and other analysis. A ceRNA regulatory network related to secondary hair follicle development was identified, which verifies the existence of circRNA5712, circRNA2109, circRNA9127, and circRNA3691 in the ceRNA regulatory network. The functional analysis showed that there was a targeting relationship between circRNA5712 and chi-miR-27b-3p and chi-miR-27b-3p and DLL4, which provided the basis for the subsequent in-depth analysis of the regulatory network of circRNA5712-chi-miR-27b-3p-DLL4 in cashmere goats. The molecular mechanism of hair follicle development has laid an important foundation.

## Data availability statement

The original contributions presented in the study are included in the article/Supplementary material, further inquiries can be directed to the corresponding author/s.

## Ethics statement

The animal study was reviewed and approved by Approved by the Scientific Research and Academic Ethics Committee of Inner Mongolia Agricultural University and the Biomedical Research Ethics of Inner Mongolia Agricultural University (Approval No. [2020] 056).

## Author contributions

FS, RM, and YZhan conceived the idea and designed the study. FS, SN, YL, YQ, MW, ZhixW, and RW participated in sample collection. FS, RM, JP, and YR performed the experiments. FS, ZhiyW, QL, RS, ZL, and YZhao analyzed the data. FS wrote the draft. FS, YZhan, and JL finalized the manuscript. All authors read and approved the final manuscript.

## Funding

This reported work was supported by the National Key R&D Program of China (2021YFD1200902), Major Science and Technology Program of Inner Mongolia Autonomous Region (2021ZD0012), and Postgraduate Scientific Research Innovation Project (B20210175Z). The funding played a role in the design of the study, the collection of data, and completion of the test.

## Conflict of interest

The authors declare that the research was conducted in the absence of any commercial or financial relationships that could be construed as a potential conflict of interest.

## Publisher's note

All claims expressed in this article are solely those of the authors and do not necessarily represent those of their affiliated organizations, or those of the publisher, the editors and the reviewers. Any product that may be evaluated in this article, or claim that may be made by its manufacturer, is not guaranteed or endorsed by the publisher.
