# Supplementary material for: Construction and functional analysis of ceRNA regulatory network related to the development of secondary hair follicles in Inner Mongolia cashmere goats
Source: Front Vet Sci. 2022 Aug 25;9:959952. doi: 10.3389/fvets.2022.959952 (PMC9453165; doi:10.3389/fvets.2022.959952)
Supplement: Supplementary file 2 [file Table_2.DOCX]

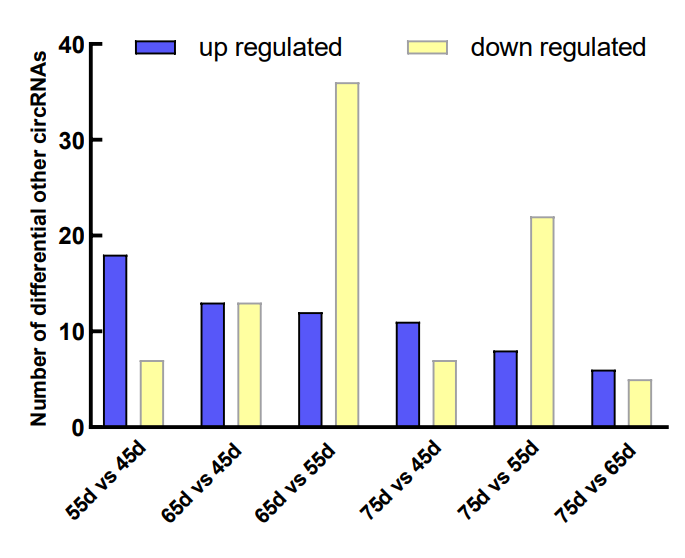
 **Additional files 2：Figure S2** Differentially other circRNAs in different groups.The purple column represents up-regulated circRNA and the yellow column represents down-regulated.
