# Supplementary material for: Construction and functional analysis of ceRNA regulatory network related to the development of secondary hair follicles in Inner Mongolia cashmere goats
Source: Front Vet Sci. 2022 Aug 25;9:959952. doi: 10.3389/fvets.2022.959952 (PMC9453165; doi:10.3389/fvets.2022.959952)
Supplement: Supplementary file 3 [file Table_3.DOCX]

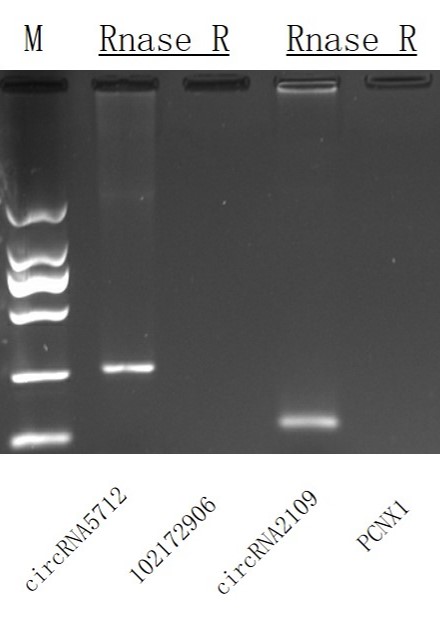

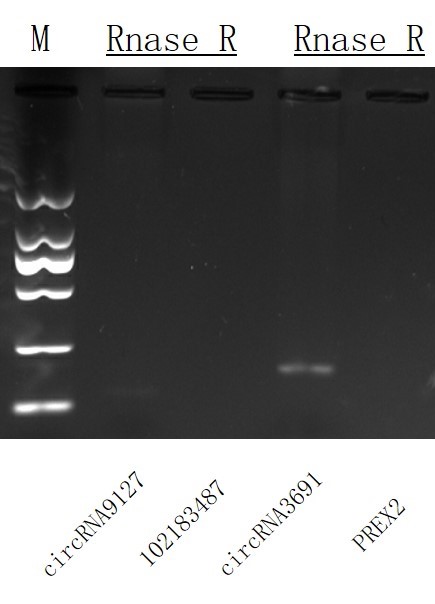


**Additional files 3：Figure S3** R Nase R treated total RNA and detected circRNA and its corresponding host genes
